# Correction: Organic–inorganic hexahalometalate-crystal semiconductor K_2_(Sn, Se, Te)Br_6_ hybrid double perovskites for solar energy applications

**DOI:** 10.1039/d5ra90062j

**Published:** 2025-05-23

**Authors:** K. Bouferrache, M. A. Ghebouli, B. Ghebouli, M. Fatmi, Sameh I. Ahmed

**Affiliations:** a Research Unit on Emerging Materials (RUEM), University Ferhat Abbas of Setif 1 Setif 19000 Algeria fatmimessaoud@yahoo.fr; b Department of Physics, Faculty of Sciences, University of M’sila University Pole Road Bourdj Bou Arreiridj 28000 M’sila Algeria; c Department of Chemistry, Faculty of Sciences, University of M’sila University Pole Road Bourdj Bou Arreiridj 28000 M’sila Algeria; d Laboratory for the Study of Surfaces and Interfaces of Solid Materials (LESIMS), University Ferhat Abbas of Setif 1 Setif 19000 Algeria; e Department of Physics, College of Sciences, Taif University P. O. Box 11099 Taif 21944 Saudi Arabia a.sameh@tu.edu.sa

## Abstract

Correction for ‘Organic–inorganic hexahalometalate-crystal semiconductor K_2_(Sn, Se, Te)Br_6_ hybrid double perovskites for solar energy applications’ by K. Bouferrache *et al.*, *RSC Adv.*, 2025, **15**, 11923–11933, https://doi.org/10.1039/D5RA00862J.

The authors regret that the email address for one of the corresponding authors, Sameh I. Ahmed, was incorrectly shown in the original manuscript. The correct email address is as shown here.

The Royal Society of Chemistry apologises for these errors and any consequent inconvenience to authors and readers.

